# Histone acetylation: a requirement for petunia floral scent

**DOI:** 10.1093/jxb/erab092

**Published:** 2021-05-04

**Authors:** Konstantinos E Vlachonasios

**Affiliations:** 1 Department of Botany, School of Biology, Aristotle University of Thessaloniki, Greece; 2 Natural Products Research Centre of Excellence (NatPro-AUTh), Center of Interdisciplinary Research and Innovation of Aristotle University of Thessaloniki (CIRI-AUTh), Thessaloniki, Greece

**Keywords:** Benzenoid, chromatin, ELP3, floral scent, GCN5, histone acetylation, histone acetyltrasferase, H3K9ac, H3K14ac, phenylpropanoid, VOC

## Abstract

This article comments on:

**Patrick RM, Huang X-Q, Dudareva N, Li Y.** 2021. Dynamic histone acetylation in floral volatile synthesis and emission in petunia flowers. Journal of Experimental Botany **72**, 3704–3722.

**Biosynthesis of volatile organic compounds (VOCs) in plants depends on the biosynthetic pathways of the primary metabolite. These regulations require molecular coordination at different levels on both primary and secondary metabolic networks by specific transcription factors that temporarily activate transcription and scent emission in plant flowers. However, very little is known about the epigenetic regulation of VOC biosynthetic genes. Now, Patrick *et al.* (2021) report that H3K9 acetylation is required for proper gene expression of primary and secondary metabolite pathways involved in petunia floral scent. This work reveals that chromatin regulatory mechanisms are essential for activation of VOC biosynthesis in petunia flowers.**

Plants synthesize a plethora of secondary metabolites that offer a wide range of functions, including plant growth, development, and defence against biotic and abiotic environments (Pichersky *et al.*, 2006). One subset of those metabolites consists of VOCs classified in four major groups: terpenoids, phenylpropanoids/benzenoids, fatty acids, and carotenoid derivatives, as well as nitrogen- or sulfur-containing compounds (Schuurink *et al.*, 2006). Plants use VOCs for communication and interaction with the surrounding environment, including attraction of pollinators and seed dispersers, defence against biotic factors and abiotic stresses, and for plant–plant signalling (Picazo-Aragonés *et al.*, 2020).

The flowers of *Petunia hybrida* cv. Mitchell emit high levels of phenylalanine-derived phenylpropanoid/benzenoid acid volatiles in the evening. Therefore, petunia has emerged as a model system to study volatile benzenoid synthesis, emission, and regulation (Dudareva *et al.*, 2013). Phe synthesis occur via the shikimate pathway that connects carbon metabolism to Phe (Maeda and Dudareva, 2012). The major volatile phenylpropanoid (C_6_–C_3_) and benzenoid (C_6_–C_1_) compounds are produced from catalysis of Phe to *trans*-cinnamic acid through l-phenylalanine ammonia-lyase (PAL) action. The formation of the volatile phenylpropanoid-related (C_6_–C_2_) compounds originates directly from Phe (Dudareva *et al.*, 2013). VOC synthesis differs throughout plant development due to the differential gene expression patterns in distinct cell types (Picazo-Aragonés *et al.*, 2020). Genes involved in the synthesis of VOCs exhibit transcriptional activation that coincides with the VOC emission (Colquhoun *et al.*, 2010). Several R2R3-type MYB transcription factors control phenylpropanoid/benzenoid biosynthetic genes (Liu *et al.*, 2015). Suppression of *ODORANT 1* (*ODO1*) and *EMISSION OF BENZENOIDS I* and *II* (*EOBI* and *EOBII*) in petunia down-regulates the expression levels of many VOC biosynthetic genes and, as a result, a decrease in the emission of most volatiles was observed (Liu *et al.*, 2015). However, knowledge on the mechanisms that regulate the transcriptional reprogramming of VOC biosynthesis is still limited.

Patrick *et al.* (2021) demonstrated that histone acetylation regulates volatile phenylpropanoid/benzenoid biosynthesis pathway genes. Using ChIP-seq analysis in second day post-anthesis petunia flowers, it was found that histone H3K9 acetylation levels were elevated on many genes that are involved in the shikimate, general phenylpropanoid, and volatile phenylpropanoid/benzenoid pathways. Moreover, several genes involved in monolignol biosynthesis and eugenol/isoeugenol synthesis also have higher histone H3 acetylation levels. The increased histone H3K9 acetylation levels were in turn associated with gene activation during anthesis. Therefore, the genes involved in the shikimate and Phe biosynthetic pathway are a target of histone H3K9 acetylation, and the biosynthesis of VOCs is regulated at both the epigenetic and transcriptional level.

Histone acetylation neutralizes the positively charged Lys residues on histone tails. As a result, the interaction between DNA and histones weakens, making chromatin more accessible to transcription factors. The transcription factors will activate or repress gene expression (Roth *et al.*, 2001). Histone acetylation is catalysed by histone acetyltransferases (HATs). In eukaryotes, four types of nuclear-localized HATs have described. The GNAT (GCN5-related N-terminal acetyltransferase), MYST (MOZ, Ybf2/SAS3, SAS2, and Tip60-related), p300/CREB-binding protein (CBP), and TAF1 (TATA-binding associated factor) families. In Arabidopsis, three GNAT-type proteins—General Control Nonderepressible 5 (GCN5, also known as HAG1), Elongator complex protein 3 (ELP3 also known as HAG3), and HAG2; two MYST types—HAM1 and HAM2 (known also as, HAG4 and HAG5); five p300/CBP types—HAC1, HAC2, HAC4, HAC5, and HAC12; and two TAF1 types—HAF1 and HAF2 (Pandey *et al.*, 2002)—are known. Multiple HATs are responsible for H3K9 acetylation, while several HATs such as GCN5 specifically acetylate H3K14. Besides histones, GCN5 and other HATs could also acetylate numerous proteins (Sterner and Berger, 2000). The enzymatic specificity of the HATs reflects their specific role in the regulation of gene expression, as acetylated Lys of histones is recognized by bromodomain-containing proteins. These histone modification readers can cause diverse epigenetic consequences (Marmorstein and Zhou, 2014).

Histone acetylation modulates chromatin dynamics in plant responses to internal and external cues to regulate downstream gene expression as signalling outputs (Jiang *et al.*, 2020). Furthermore, histone acetylation affects multiple processes that span the entire life cycle, including specific metabolic or developmental processes and adaptation to environmental cues (Grasser *et al.*, 2020) (Box 1).

The histone acetylation and the transcriptomic analysis on the second day after anthesis of petunia flower points toward the molecular processes that contribute to VOC biosynthetic genes. This work could be used as a reference point to reveal the role of histone modifications in coordination of terpenoid biosynthesis genes or volatile, fatty acid derivative compounds in other plants. Epigenetic factors are relevant modulators of rapid responses to the environment, enabling plants to adapt to stress events more efficiently and preparing the offspring for future challenges. Moreover, volatile secondary metabolites are essential for plant biotechnological applications including food, health, and industrial products, and the perfume industry (Plasmeijer *et al.*, 2020). The regulation of VOC biosynthesis at the chromatin level will have many metabolic engineering implications. For instance, the use of transgenes with the local chromatin environment that could be turned on or off by histone acetylation will facilitate the optimum regulatory circuits to produce high-level specialized metabolites.

**Box 1. Possible histone acetylation mechanisms in the VOC biosynthetic pathway in petunia**

The regulation of VOCs and the release of petunia floral scent are mediated by histone modification. Furthermore, H3K9 acetylation is correlated with transcription activation of VOC biosynthetic genes (Patrick *et al.*, 2021). Histone H3K9 acetylation is detected in genes involved in almost all steps of the shikimate pathway (blue), the primary steps of VOC biosynthesis. Moreover, genes (blue arrows) involved in the first steps of Phe-derived phenylpropanoid/benzenoid volatile compounds also received a histone acetylation mark. Therefore, in the first 2 d of open petunia flowers, several protein complexes that contain HATs should be recruited to the promoter and the loci of those genes. One of them could be the elongator complex that includes the ELP3/HAG3 acetyltransferase, since ELP3 is expressed during this developmental stage (Patrick *et al.*, 2021). In Arabidopsis, ELP3 is involved in RNAPII transcription elongation through acetylation of H3K14 in the coding and 3’-untranslated regions of specific genes (Nelissen *et al.*, 2010). Several other HAT-containing complexes, such as GCN5 or HAC, could also be recruited to the loci of VOC biosynthetic genes. In response to internal or external stimuli, specific HATs and the associated proteins are recruited to the promoter region of the responsive genes by particular transcription factors (Grasser *et al.*, 2020). The HAT complex action will trigger histone acetylation (H3K9, H3K14, H3K23, H3K27, H4, and others), which is followed by transcription activation or repression. The question is what kind of signals recruit the HAT complex into promoters of the VOC biosynthesis genes.

One possibility is the regulation of the rhythmic release of VOCs by transcription factors encoded by clock genes (Dudareva *et al.*, 2013). In petunia, the morning component LATE ELONGATED HYPOCOTYL (LHY) controls the daily expression of many VOC biosynthetic genes and specific transcription factors such as ODO1, by restricting their expression in the evening (Fenske *et al.*, 2015). Acetylation and deacetylation of the H3 histone of the clock genes could change chromatin accessibility and subsequently affect the expression of the VOC biosynthetic genes and the emission of VOCs.

Another scenario is that specific transcription factors, such as ODO1, EOBI, and EOBII, recruit HAT complexes to the promoters of genes involved in the phenylpropanoid/benzenoid biosynthetic pathway independently of circadian rhythms. During flowering development in Arabidopsis, histone acetylation and several HATs play a significant role by controlling hormone responses and orchestrating gene expression profiles in several floral organs (Poulios and Vlachonasios, 2018; Grasser *et al.*, 2020). Furthermore, ethylene regulates VOC emission after pollination in petunia by controlling several VOC genes (Underwood *et al.*, 2005). Ethylene treatment increases H3 acetylation levels on ethylene-responsive genes in Arabidopsis seedlings (Wang and Qiao, 2019). The specificity of modulation of the histone acetylation response to produce different VOCs relies on the partner(s) of the HAT complex(es). Identification of the HAT-containing complex in primary and secondary metabolic networks will reveal details of the molecular mechanism that underlies VOC biosynthesis of flowers.

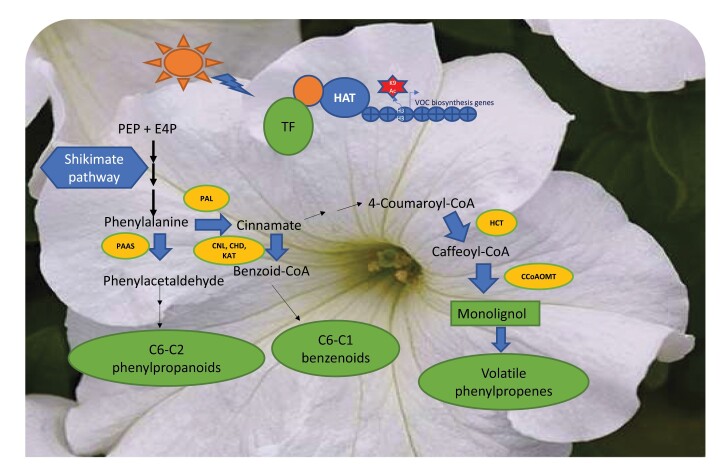

## References

[CIT0001] Colquhoun TA , Verdonk JC, Schimmel BC, Tieman DM, Underwood BA, Clark DG. 2010. Petunia floral volatile benzenoid/phenylpropanoid genes are regulated in a similar manner. Phytochemistry 71, 158–167. 19889429 10.1016/j.phytochem.2009.09.036

[CIT0002] Dudareva N , Klempien A, Muhlemann JK, Kaplan I. 2013. Biosynthesis, function and metabolic engineering of plant volatile organic compounds. New Phytologist 198, 16–32. 10.1111/nph.12145 23383981

[CIT0003] Fenske MP , Hazelton KDH, Hempton AK, Shim JS, Yamamoto BM, Riffell JA, Imaizumi T. 2015. Circadian clock gene LATE ELONGATED HYPOCOTYL directly regulates the timing of floral scent emission in Petunia. Proceedings of the National Academy of Sciences, USA 112, 9775–9780. 10.1073/pnas.1422875112 PMC4534231 26124104

[CIT0004] Grasser KD , Rubio V, Barneche F. 2020. Multifaceted activities of the plant SAGA complex. Biochimica et Biophysica Acta 1864, 194613. 32745625 10.1016/j.bbagrm.2020.194613

[CIT0005] Jiang J , Ding AB, Liu F, Zhong X. 2020. Linking signaling pathways to histone acetylation dynamics in plants. Journal of Experimental Botany 71, 5179–5190. 32333777 10.1093/jxb/eraa202 PMC7475247

[CIT0006] Liu J , Osbourn A, Ma P. 2015. MYB transcription factors as regulators of phenylpropanoid metabolism in plants. Molecular Plant 8, 689–708. 25840349 10.1016/j.molp.2015.03.012

[CIT0007] Maeda H , Dudareva N. 2012. The shikimate pathway and aromatic amino acid biosynthesis in plants. Annual Review of Plant Biology 63, 73–105. 10.1146/annurev-arplant-042811-105439 22554242

[CIT0008] Marmorstein R , Zhou MM. 2014. Writers and readers of histone acetylation: structure, mechanism, and inhibition. Cold Spring Harbor Perspectives in Biology 6, a018762. 24984779 10.1101/cshperspect.a018762 PMC4067988

[CIT0009] Nelissen H , De Groeve S, Fleury D, et al 2010. Plant elongator regulates auxin-related genes during RNA polymerase II transcription elongation. Proceedings of the National Academy of Sciences, USA 107, 1678–1683. 10.1073/pnas.0913559107 PMC2824411 20080602

[CIT0010] Pandey R , Müller A, Napoli CA, Selinger DA, Pikaard CS, Richards EJ, Bender J, Mount DW, Jorgensen RA. 2002. Analysis of histone acetyltransferase and histone deacetylase families of *Arabidopsis thaliana* suggests functional diversification of chromatin modification among multicellular eukaryotes. Nucleic Acids Research 30, 5036–5055. 12466527 10.1093/nar/gkf660 PMC137973

[CIT0011] Patrick RM , Huang ΧQ, Dudareva N, Li Y. 2021. Dynamic histone acetylation in floral volatile synthesis and emission in petunia flowers. Journal of Experimental Botany 72, 3704–3722 33606881 10.1093/jxb/erab072 PMC8096599

[CIT0012] Picazo-Aragonés J , Terrab A, Balao F. 2020. Plant volatile organic compounds evolution: transcriptional regulation, epigenetics and polyploidy. International Journal of Molecular Sciences 21, 8956. 10.3390/ijms21238956 PMC7728353 33255749

[CIT0013] Pichersky E , Noel JP, Dudareva N. 2006. Biosynthesis of plant volatiles: nature’s diversity and ingenuity. Science 311, 808–811. 16469917 10.1126/science.1118510 PMC2861909

[CIT0014] Plasmeijer M , Liao P, Haring MA, Dudareva N, Schuurink RC. 2020. Metabolic engineering of plant volatiles floral scent, flavor, defense. In: Pichersky E, Dudareva N, eds. Biology of plant volatiles. Boca Rotan, FL: CRC Press, Taylor & Francis Group LLC, 379–403.

[CIT0015] Poulios S , Vlachonasios KE. 2018. Synergistic action of GCN5 and CLAVATA1 in the regulation of gynoecium development in *Arabidopsis thaliana*. New Phytologist 220, 593–608. 10.1111/nph.15303 30027613

[CIT0016] Roth SY , Denu JM, Allis CD. 2001. Histone acetyltransferases. Annual Review of Biochemistry 70, 81–120. 10.1146/annurev.biochem.70.1.81 11395403

[CIT0017] Schuurink RC , Haring MA, Clark DG. 2006. Regulation of volatile benzenoid biosynthesis in petunia flowers. Trends in Plant Science 11, 20–25. 16226052 10.1016/j.tplants.2005.09.009

[CIT0018] Sterner DE , Berger SL. 2000. Acetylation of histones and transcription-related factors. Microbiology and Molecular Biology Reviews 64, 435–459. 10839822 10.1128/mmbr.64.2.435-459.2000 PMC98999

[CIT0019] Underwood BA , Tieman DM, Shibuya K, Dexter RJ, Loucas HM, Simkin AJ, Sims CA, Schmelz EA, Klee HJ, Clark DG. 2005. Ethylene-regulated floral volatile synthesis in petunia corollas. Plant Physiology 138, 255–266. 15849311 10.1104/pp.104.051144 PMC1104180

[CIT0020] Wang L , Qiao H. 2019. New insights in transcriptional regulation of the ethylene response in Arabidopsis. Frontiers in Plant Science 10, 790. 31275338 10.3389/fpls.2019.00790 PMC6591485

